# Visualizing the 3D Evolution and Morphology of Hydrogen-Assisted Ductile Crack Growth in Hydrogen-Precharged P355NH Steel Using X-Ray Micro-Computed Tomography

**DOI:** 10.3390/ma19071335

**Published:** 2026-03-27

**Authors:** Alexander Hell, Jonas Fell, Torben Werning, Hans-Georg Herrmann

**Affiliations:** 1 Lightweight Systems, Department of Materials Science and Engineering, Saarland University, 66123 Saarbrücken, Germanyhans-georg.herrmann@izfp.fraunhofer.de (H.-G.H.); 2 Core Facility for Correlative Microscopy and Tomography CoMiTo, Faculty of Natural Sciences and Technology, 66123 Saarbrücken, Germany; jonas.fell@uni-saarland.de; 3 Fraunhofer Institute for Nondestructive Testing IZFP, 66123 Saarbrücken, Germany

**Keywords:** hydrogen-assisted crack growth, fracture mechanical testing, micro-computed tomography

## Abstract

Hydrogen embrittlement is known to adversely affect the mechanical properties of low-carbon steels used for pipelines and pressure vessels, leading to accelerated crack growth and lowered fracture toughness. To overcome the limitations of surface-based analysis, this study employs X-ray micro-computed tomography (µ-CT) to provide a comprehensive 3D evaluation of the crack evolution. This approach is used to assess hydrogen-assisted crack growth in P355NH compact tension samples from previous fracture mechanical tests and enables a precise quantification of the internal crack path and the crack tip opening angle (CTOA) across the entire specimen thickness as well as the local damage morphology. By integrating these spatial parameters, a deeper understanding of the impact of hydrogen on local fracture mechanisms is achieved, revealing insights that have remained hidden in previous two-dimensional microscopy observations. For instance, µ-CT results clearly demonstrate that the hydrogen-assisted crack propagation is associated with increased void formation and secondary cracking in vicinity of the crack tip. However, it is proposed that the results are superimposed with continuous hydrogen desorption, which implies a need for in situ charging during mechanical loading and an analysis of the hydrogen concentration profile. Both will be the scope of further studies.

## 1. Introduction

X-ray tomography has a long history of application in non-destructive testing. It enables three-dimensional quality assessment and damage identification in technical components through the penetration, weakening and scattering of X-rays in matter [1]. Micro-computed tomography (µ-CT) allows 3D and through-thickness imaging of structures with a high spatial resolution on the microscale. Therefore, X-ray μ-CT has become an essential technique for capturing the three-dimensional evolution of internal damage and crack morphology [2,3,4,5].

Within the transition from fossil fuels to renewable energy sources, green hydrogen has the potential for enhancing decarbonization of transportation and industry [6]. However, the adverse effect of hydrogen environments on the failure properties of metal components, known as hydrogen embrittlement (HE), still poses a challenge [7]. Low-carbon steels, which are typically used for gas pipelines and pressure vessels, may suffer from a degradation of fracture toughness and fatigue crack growth properties in the presence of hydrogen [8,9].

Scanning electron microscopy (SEM) enables the characterization of microstructural effects responsible for HE on fracture surfaces. Morphologic characteristics revealed via SEM fractography of steels undergoing HE involve unaffected or altered ductile micro-void structures as well as transgranular quasi-cleavage and intergranular cleavage [10]. Traditionally, microscopic investigations on 2D cross-sections have been an important method for studying hydrogen-influenced cracking, such as the example displayed in [11]. However, these planar observations often fail to represent the true volumetric nature of the crack propagation and damage morphology. Tomographic information can be acquired for instance via a focused ion beam FIB/SEM serial sectioning approach [12], but only for micro-sized sample volumes.

Beyond the well-established capabilities of SEM analysis, X-ray tomography enables the three-dimensional characterization of hydrogen-assisted crack growth, offering a volumetric perspective on hydrogen-related crack propagation [12]. In high strength martensitic steel, X-ray CT enabled the characterization of macroscopic crack propagation, with hydrogen-charged samples exhibiting more continuous crack growth with a lower crack opening than in the uncharged state [12]. The CT investigation was performed using thin sample geometries extracted from the mid-thickness regions of compact tension specimens [12]. Via µ-CT, subsequent studies were allowed to resolve the impact of different hydrogen concentrations on crack morphology as well as a more in-depth correlation of cracking mechanisms and a lowered crack growth resistance due to hydrogen [13]. It was revealed that the J-integral as a fracture parameter decreased in hydrogen-charged specimens. This was attributed to the more continuous and sharper crack front compared to uncharged samples, where crack meandering and branching frequently occurred, involving prior austenite grain boundaries and consuming more fracture energy [13]. Furthermore, µ-CT has been implemented as a tool to gain understanding of hydrogen-influenced ductile damage progression and fracture processes in pipeline steel. Smooth and notched round bar tensile samples were machined of grade X70 in [14] to assess the impact of stress triaxiality on failure in the presence of hydrogen with severe embrittlement occurring for very sharp notches. Using µ-CT, it was found that cracking with respect to L-T and L-S orientation occurred preferentially in T-direction when L is the loading axis and hydrogen facilitates void nucleation—but seemingly not void volume growth—even at low strains [14]. However, an effect on the void network was not perceivable for X56 pipeline steel in similar µ-CT studies [15], from which it was concluded that mechanical and microstructural material characteristics play an important role for the effect of hydrogen on 3D damage mechanisms. For instance, X56 has a lower strength than X70. Furthermore, the concept of a critical hydrogen concentration, which has to be reached before fundamental alterations in the damage behavior occur, was cited [15]. Hydrogen-induced cracks in austenitic stainless steels prone to deformation-induced martensite transformation were analyzed via µ-CT in [16]. Although a lab-based approach with a 160 kV nano-focus tube has proven to be challenging for the investigation of smaller cracks, crack tips or alloy element segregation, the authors point out the advantages of being able to observe the entire volumetric crack front in one analysis and also suggest the application of synchrotron radiation for better signal [16]. Micro-computed synchrotron tomography on X65 and X60 pipeline steel was performed in [17] to assess hydrogen-induced cracks after charging; blisters and the damage morphology in in situ- and ex situ-charged slow strain rate tensile tests. Failure initiation as well as progression and fracture mode (brittle and ductile fracture) were studied, with the authors specifically citing the benefits over a 2D microscopic analysis, e.g., in terms of potential misconceptions [17]. The need for comprehensive 3D tomographic investigation in HE studies is therefore clearly recognizable. In this context, synchrotron-based X-ray refraction techniques can further complement the information from absorption-based synchrotron µ-CT as demonstrated in [18]. Synchrotron X-ray refraction CT greatly enhanced contrast and enabled robust defect segmentation with a better detectability of microcracks compared to absorption-based synchrotron CT [18].

Bridging the gap to fracture mechanics, this study relies on micro-computed tomography for a detailed investigation of crack propagation and morphology in fracture mechanical samples made of uncharged and hydrogen-precharged ferritic pressure vessel steel. Large volume scans of crack fronts in compact tension specimens were performed, supported by CT analysis of local cracking behavior via extraction of small samples from the original macroscopic crack. The findings from X-ray tomographic analysis are related to the results from previous quasi-static fracture mechanical tests and SEM fractography (see [19]). By combining μ-CT reconstructions of the entire crack front in compact tension specimens with high-resolution scans of specific regions of interest (ROI), it becomes evident how internal hydrogen affects fracture behavior. This multiscale analysis reveals the impact of hydrogen precharging and subsequent desorption on ductile crack propagation and local void evolution. Furthermore, an outlook is given on how µ-CT-application for the analysis of hydrogen-assisted crack growth could be enhanced in future works as the study also briefly addresses the challenges linked to the high X-ray absorption of steel.

## 2. Materials and Methods

### 2.1. Steel Selection and Sample Preparation for Mechanical Testing

**Figure 1:**
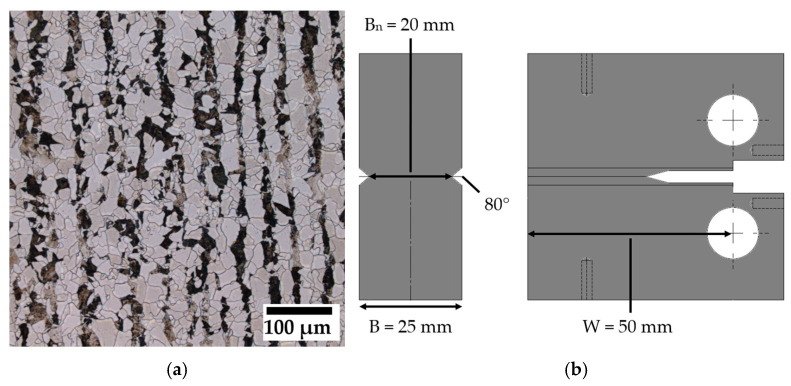
(**a**) P355NH microstructure and (**b**) manufactured compact tension specimen geometry in orientation L-T (denoted as CT50 in the following).

Specimens for fracture mechanical testing were manufactured from rolled low-alloy pressure vessel steel P355NH. In optical micrographs, P355NH exhibits a fine grain ferritic–pearlitic microstructure and a rolling texture (Figure 1a). Compact tension specimens (CT50) with a width W = 50 mm and thickness B = 25 mm were machined with the notch perpendicular to the rolling direction as observed in Figure 1a (L-T direction) using conventional manufacturing processes and wire-cut electric discharge machining (EDM). The samples were side grooved to a net thickness of B_n_ = 20 mm to increase stress triaxiality and guide the crack fronts (see Figure 1b for complete specimen geometry).

### 2.2. Hydrogen Precharging and Fracture Mechanical Experiments

Hydrogen precharging experiments of the CT50 specimens were performed using a cathodic hydrogen charging process with 0.25 mol/L sulphuric acid. The specimens were precharged for a duration of four days with a current density of 1.2 mA/cm^2^. Potassium iodide was used as a recombination inhibitor. Until mechanical testing was performed, the specimens were stored in liquid nitrogen to prevent excessive hydrogen desorption. More details regarding sample preparation and charging conditions can be found in [19].

Rising displacement J-Δa-testing was performed for P355NH (see [19] for a description of methodology and the fracture mechanical results). The fatigue precracking procedure aimed at rapid crack generation to minimize the time for hydrogen desorption before subsequent J-Δa-testing. As shown in [19], sinusoidal load-controlled cycling with a frequency of 10 Hz, R = 0.1 and a peak force of 19 kN leads to precracks with a sufficient size of 0.5 W after approximately 30–45 min and ensures small-scale yielding conditions for P355NH. Fracture surfaces and 2D metallographic cross-sections of selected specimens were examined via SEM in the previous work [19]. In the present study, a total of three uncharged and three hydrogen-precharged compact tension samples undergo a 3D investigation using X-ray micro-computed tomography.

### 2.3. Manufacturing of Specimens for High-Resolution µ-CT Scans

First, µ-CT scans were performed on the CT50 specimens after fracture mechanical testing to image the entire crack front. However, due to the high X-ray absorption of steel, smaller ROI volumes were machined from the CT50 specimens afterwards. This reduction in volume was necessary to achieve high spatial resolution of less than 10 µm and to accurately capture the internal void network. Using a wire-cut electric discharge machining (EDM) MV 2400S system (Mitsubishi Electric Europe B.V., Ratingen, Germany), ROI samples with a thickness of 1 mm containing ductile crack front, fatigue crack surface and notch tip were extracted from the compact tension specimens after fracture mechanical testing. To minimize excessive thermal effects and residual stress during specimen preparation, the samples were carefully machined with optimum water cooling and a thin EDM wire.

**Figure 2:**
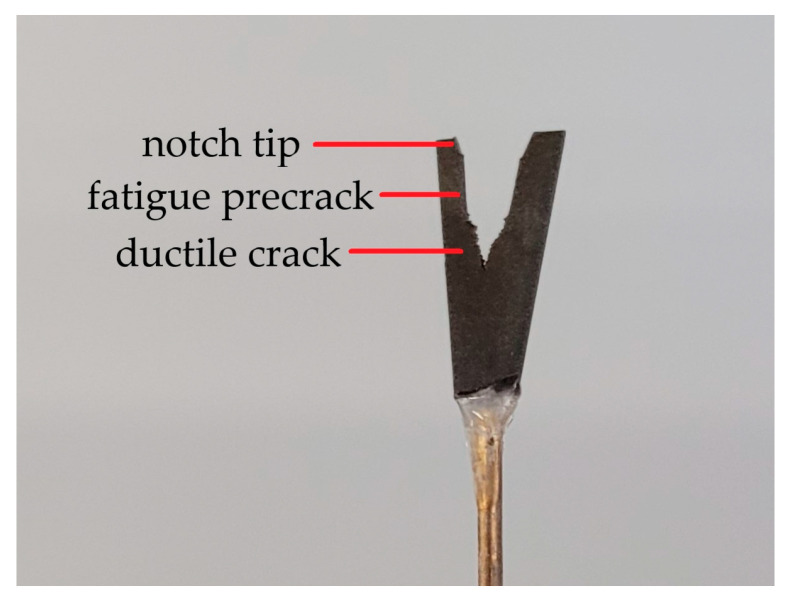
ROI sample for high-resolution µ-CT scans glued to a rod-shaped specimen holder. The sample contains notch tip, fatigue precrack and ductile crack front.

The ROI volumes were machined from two CT50 specimens of each condition—uncharged and hydrogen precharged. One ROI sample was extracted from the center section of the CT50 specimens (mid-thickness) and two additional ones were machined near the outer edges or side grooves, respectively, resulting in a total number of six ROI volumes per charging condition. The crack slices were glued to a specimen holder for µ-CT scans (Figure 2).

### 2.4. µ-CT-Characterization and Analysis

For micro-computed X-ray tomography, an EasyTom L system from RX-Solutions (Chavanod, France) was used. The device was equipped with a 230 kV micro-focus reflection X-ray tube with a maximum of 200 W target power. The detector was a VIVIX-V 2532D from Vieworks (Anyang, Republic of Korea) with a pixel size of 124 µm and an active pixel array of 2048 × 2560 px. Table 1 summarizes the scan settings for the investigation of the different sample geometries.

**Figure 3:**
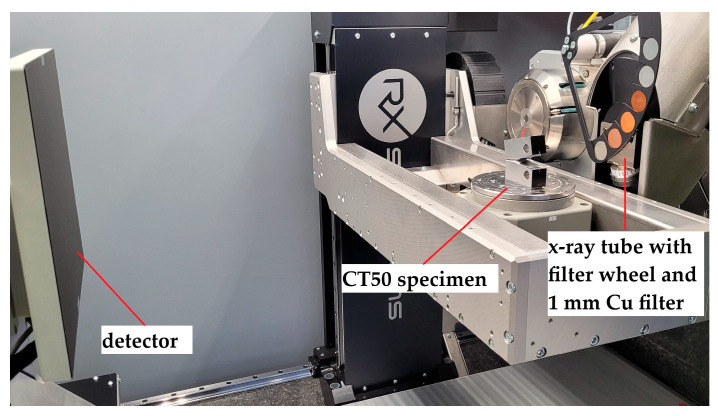
µ-CT measurement setup for compact tension CT50 specimens.

**Figure 4:**
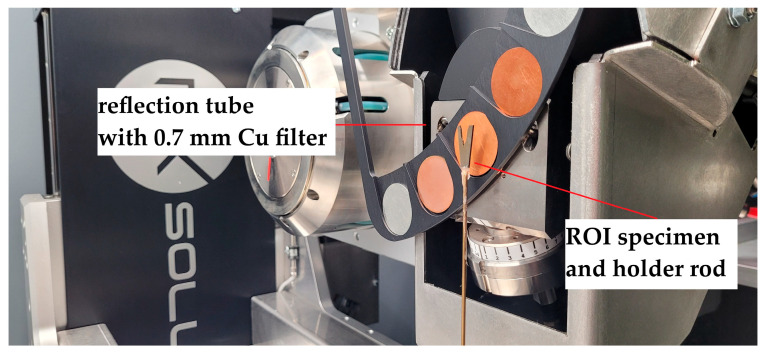
µ-CT measurement setup for ROI samples.

To achieve sufficient X-ray intensity for the analysis of crack fronts in thick CT50 specimen geometries, 230 kV acceleration voltage was applied, combined with a high target power of 55 W and 2 × 2 detector binning. However, this limits spatial resolution to a minimum voxel size of 55 µm. A tenfold reduction in voxel size is reached in the investigation of the ROI samples at 200 kV, as the specimens were adjusted closer to the tube and the target power was reduced to 7 W. To ensure high signal-to-noise ratio and reliable segmentation results in subsequent analysis, 2 × 2 binning was applied again as the low target power limits X-ray intensity. Although spatial resolution could potentially be further enhanced to up to 1.5 µm voxel size (e.g., using larger SDD, frame rate of 1 fps and omitted binning), preliminary tests have shown that the low SNR coupled with beam hardening and metal artifacts render the void segmentation increasingly unreliable. Optimal image quality was achieved with the ROI sample parameters in Table 1. Details of the segmentation methodology are described in subsequent sections. Figure 3 and Figure 4 show the µ-CT measurement setup for the characterization of the CT50 and ROI specimens, respectively.

Volumetric reconstruction was performed using a filtered back-projection algorithm implemented in the software Xact 25.04.1 (RX-Solutions) while ring and beam hardening artifacts were mitigated with appropriate filter settings. Void segmentations in the ROI specimens were performed using Dragonfly 3D World 2025.1 (Comet Technologies Canada Inc., Montréal, QC, Canada) by first applying an anisotropic diffusion filter for denoising while preserving edges in the slices. Afterwards, simple thresholding allowed conservative and robust segmentation of voids with a minimum size of 30 µm (>5 voxels) as a result of anisotropic filtering and the good SNR obtained with the scan settings in Table 1.

In contrast to the void segmentation, simple thresholding proved insufficient for the segmentation of the crack fronts in the CT50 specimens because of high X-ray attenuation causing a heavy loss of signal intensity. Strong beam hardening and metal artifacts occurred due to the large absorbing steel volume—especially in contrast to the smaller ROI samples. We therefore opted for the use of Dragonfly’s machine learning methodology via the Segmentation Wizard. For our model, the Attention U-Net architecture with the Cross-Entropy loss function and Adadelta optimization algorithm were used. The model was trained on the original gray scale CT data and a Sobel-filtered version, as the filter strongly enhances the crack edges. In addition, 2.5 D input data and data augmentation were utilized to increase the variability of the training data. The model was initially trained on a single representative slice from one specimen. Subsequently, regions where the segmentation accuracy was lower, specifically the crack tip and specimen edges, were identified and included in the training data until stable segmentation results were achieved across the full specimen. The model was validated against nine manually segmented slices of a different specimen (ground truth) selected to represent different regions of the specimen. The model achieved a Dice coefficient of approximately 99.40% and a True Positive Ratio (TPR) of 99.91% against the manually annotated ground truth. The high agreement in the validation data is attributed to deviations between prediction and ground truth being confined to the air-steel boundary, where they are typically within 1–2 voxels, which is comparable to the uncertainty of the manual selection. The trained model was subsequently applied to all specimens without retraining. Visual material illustrating the quality of the machine learning approach in comparison to a simple thresholding methodology is provided in Appendix A.

Supported by a Python code which was implemented in Dragonfly, the total crack length, segmented into ductile and fatigue crack, and the crack tip opening angle (CTOA) were determined in thickness direction of the specimens. The analysis was performed on a binary crack ROI, obtained via the machine learning segmentation model. For each slice along specimen thickness, the crack tip was defined as the outermost voxel in crack growth direction. The total crack length was then calculated as the lateral distance between this point and the notch tip, which was defined from the segmented geometry. To distinguish between the fatigue and the ductile crack, points marking the fatigue–ductile transition were visually identified along the upper and lower crack face, using the local change in surface roughness and morphology upon the transition as a reference. To minimize the uncertainty in the determination of the transition front arising from potential operator errors, a minimum of 15 points per crack flank were selected and the results averaged for the upper and lower crack face (see Appendix A).

Afterwards, the transition front along the specimen thickness direction was interpolated via piecewise cubic Hermite interpolating polynomials (PCHIP) and averaged over the upper and lower crack face. Near the zone affected by the side grooves, a differentiation between fatigue crack and ductile crack using visual identification and Python code became increasingly difficult. Therefore, the evaluation was restricted to the range defined by the first and last manually selected reliable transition points, and no extrapolation beyond these bounds was performed. In this paper, a first representative CTOA was measured as defined in [20] at 1 mm distance behind the pre-determined crack tip from the separation of the upper and lower crack face in each slice after initial measurements at different distances from the crack tip showed no significant deviations. However, the application of an alternative approach, such as the example displayed in [21], is possible in future studies. For both, ductile crack length and CTOA, a total of approximately 300 measurement points resulted across the specimen thickness. Post-processing of the measurements for each specimen was performed in OriginLab 2025b. To reduce noise in crack tip opening angle measurements, moving mean filtering with a window size of nine points was applied. A linear interpolation of the ductile crack extension Δa and CTOA values was performed with respect to the specimen coordinate to ensure comparability and to generate mean and scatter bands for the data collected.

## 3. Results

### 3.1. Summary of Mechanical Testing

The J-Δa-curves for P355NH, as reported and displayed in [19], have shown a facilitation of stable, ductile crack extension in H-precharged CT50 samples due to a lowered crack growth resistance indicated by a flattening of the J-Δa-curve. However, no unstable cracking behavior and no signs of cleavage-like failure could be found on the two-dimensional fracture surface in SEM. The dimpled fracture morphology did not exhibit any clear changes [19]. A reduced CTOA and increased ductile crack extension were observed in 2D micrographs of H-precharged specimens, but the optical microscopy did not enable any investigations of the void distribution and crack morphology in three dimensions [19]. It has been proposed that a more critical degradation of mechanical properties might occur for in situ-charged P355NH [19].

### 3.2. Volume Scans of Compact Tension Specimens Including CTOA and Crack Length Measurement

**Figure 5:**
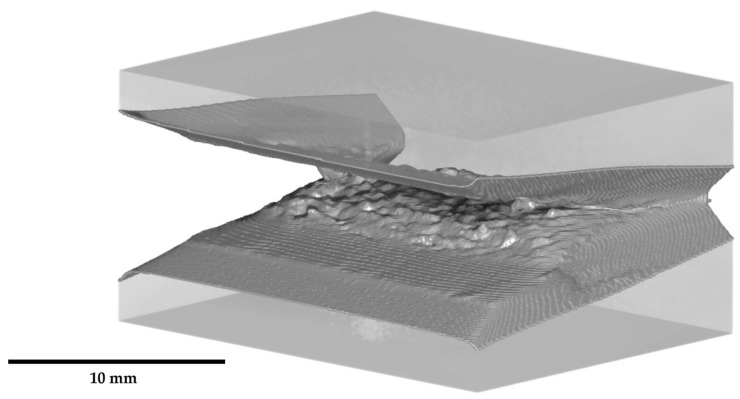
Illustration of the crack segmentation for CT50 specimens across the entire specimen thickness.

Figure 5 illustrates the segmented crack front across the entire thickness of a compact tension specimen. The 3D models clearly resolve the notch tip, the planar fatigue crack, and the characteristic roughness of the ductile crack, along with the side grooves. These volumetric reconstructions of both uncharged and hydrogen-precharged specimens form the basis for the subsequent analysis.

**Figure 6:**
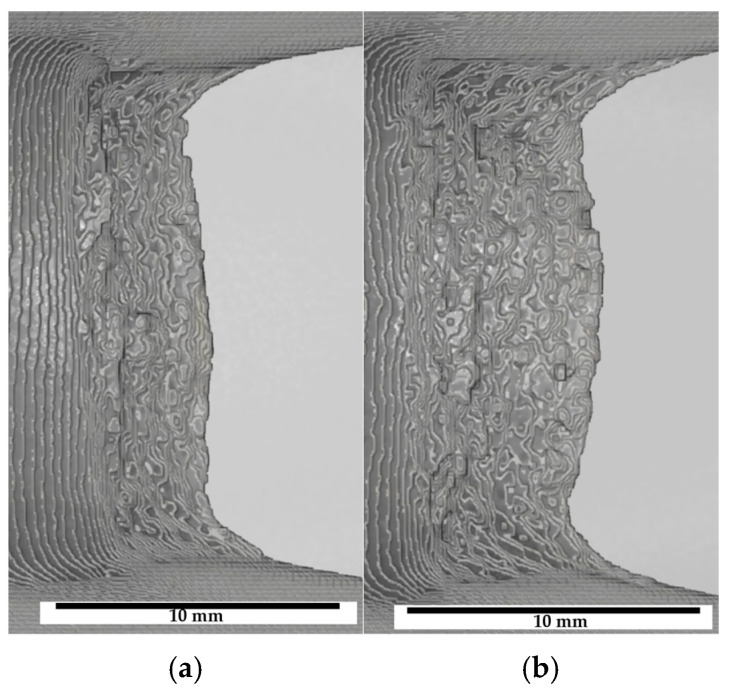
Top view of 3D models highlighting the crack surface topography. This specific visualization of the 3D models allows for an easy identification of the crack front and ductile crack curvature in (**a**) uncharged and (**b**) H-precharged P355NH after J-Δa testing.

Crack tunneling is observed in the hydrogen-precharged state when the most pronounced reduction in crack growth resistance occurred in mechanical testing. As an example, a top view of segmented crack fronts of an uncharged specimen and an H-charged one exhibiting crack tunneling is shown in Figure 6. Therefore, localized acceleration of crack growth within the specimen’s interior is indicated for the precharged condition.

**Figure 7:**
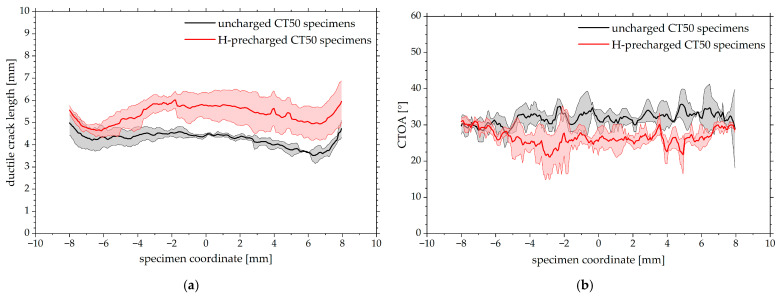
Mean and scatter bands of ductile crack length and CTOA measured across the CT50 specimen thickness direction based on tomographic reconstruction data from three individual uncharged and precharged specimens, obtained via linear interpolation of measurement data. A specimen coordinate of 0 mm corresponds to the center section of the specimen. (**a**) Ductile crack length; (**b**) crack tip opening angle (CTOA). Calculation of Δa and CTOA near the outermost edges (≥±8 mm specimen coordinate) could not be reliably established as discussed above and is therefore omitted.

The results of the ductile crack length determination as well as the CTOA measurement are displayed in Figure 7a,b. In hydrogen-precharged specimens, ductile crack fronts further extend in the material, especially at mid-thickness, emphasizing the results from visual inspection of tomographic data as shown above. Approaching the outer edges, cracks in both charging conditions are slightly longer in general possibly because of the influence of side grooves on stress triaxiality. However, the measurements align quite well with the fracture mechanical crack length calculations and result in [19], further confirming them. The evaluation of CTOA (Figure 7b) reveals a notably lower opening angle in precharged CT50 specimens, indicating a reduced ductile crack growth resistance. Further comparing the two material conditions, it is remarkable that both measured ductile crack extension and especially CTOA show the least difference near the specimen edges, with the average CTOA at ±8 mm specimen coordinate being approximately equal.

### 3.3. Characterization of Ductile Crack Morphology via High-Resolution µ-CT of ROI Samples

**Figure 8:**
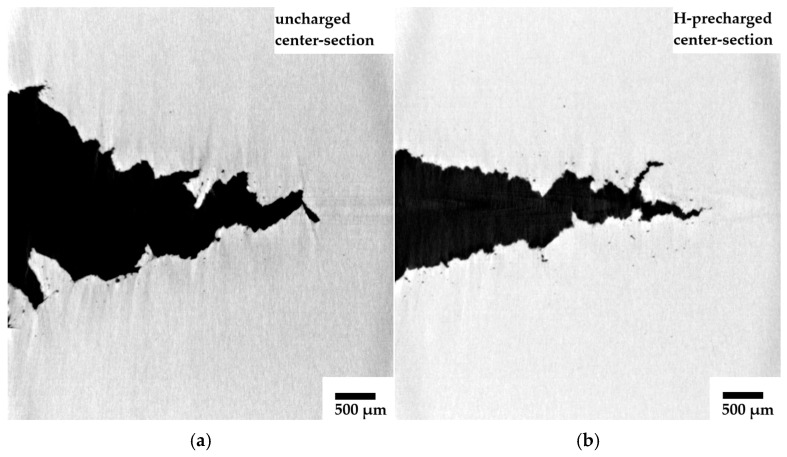

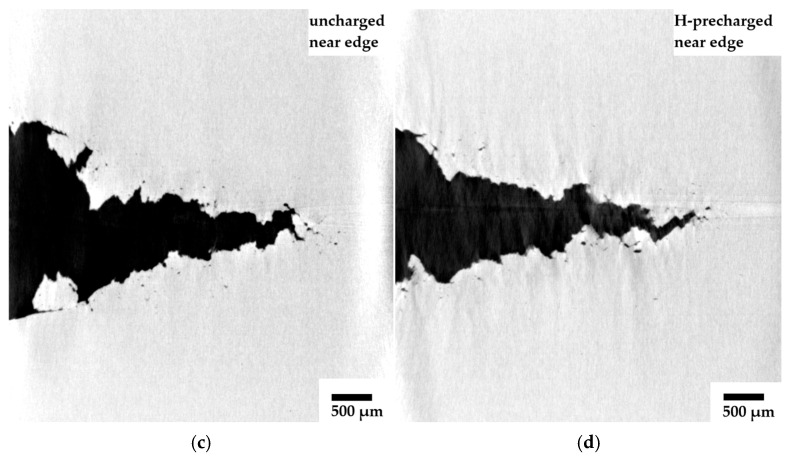
Reconstructed slices of ROI samples containing ductile crack fronts from different locations in CT50 specimens after J-Δa testing. (**a**) Uncharged condition from CT50 specimen center; (**b**) H-precharged P355NH from CT50 specimen center; (**c**) uncharged condition near CT50 edge; (**d**) H-precharged P355NH near CT50 edge.

A segmentation of the crack surfaces in the ROI sample material reveals rough fracture surfaces in both hydrogen uncharged and precharged steel, which is also typical for ductile crack growth (Figure 8a–d). Remarkably, the H-precharged condition exhibits an increased tendency for secondary cracking close to the crack tip in ROI samples extracted from the center section of the compact tension specimens (Figure 8b)—besides the obvious difference in crack opening. CT reconstruction data of specimens exhibiting secondary cracking is provided as Supplementary Material (Figures S1 and S2). However, a distinction of uncharged and hydrogen-charged samples based solely on crack morphology and sometimes even based on the opening of the crack front cannot be reliably established in samples extracted near the side grooves. Two extremes where no deviation is visible are shown, for instance, in Figure 8c,d.

### 3.4. Void Analysis for Ductile Crack Growth

**Figure 9:**
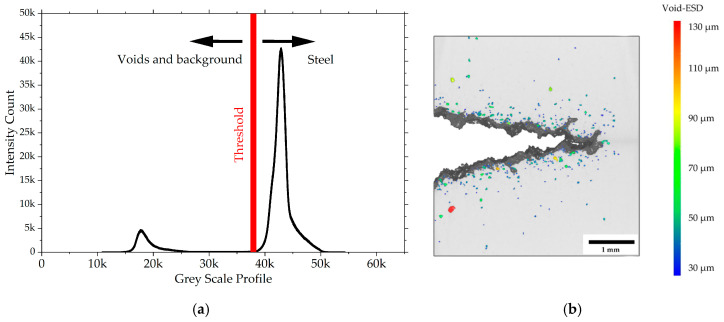
Void volume segmentation. (**a**) Illustration of the simple thresholding methodology; (**b**) side view of the 3D model with superimposed depth information showing the void distribution near the ductile crack front for hydrogen-precharged P355NH. The colormap encodes the void ESD, allowing for a clear distinction between micro-voids (blue) and larger coalesced cavities (red).

**Figure 10:**
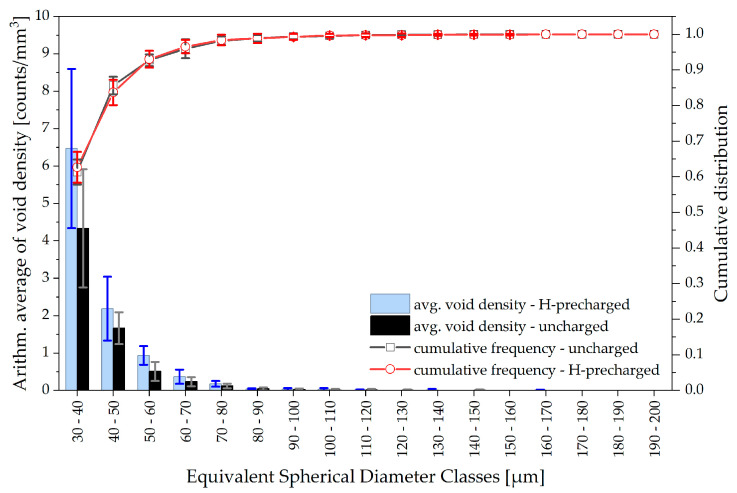
Average void densities (void count per unit sample volume) for different equivalent spherical diameter classes and averaged cumulative frequencies for hydrogen-precharged and uncharged P355NH ROI samples. A total of six ROI samples is used from two independent CT50 specimens for each charging condition.

Deformation-induced voids are segmented using simple thresholding. The process is illustrated in Figure 9. The equivalent spherical diameter (ESD) is used as a representative diameter for void classification. Voids smaller than 30 µm ESD are omitted as the thresholding segmentation is unreliable for air inclusions spanning only one to four voxels. The quantitative analysis in Figure 10 shows the arithmetic average and standard deviation of the void density, e.g., average void count per unit sample volume, for all samples investigated with respect to size classes of the ESD ranging from 30 to 200 µm (10 µm bin width). The comparison of uncharged and precharged conditions shows clear evidence that ROI samples from hydrogen-precharged CT50 specimens exhibit a significantly increased number of deformation-induced cavities.

**Figure 11:**
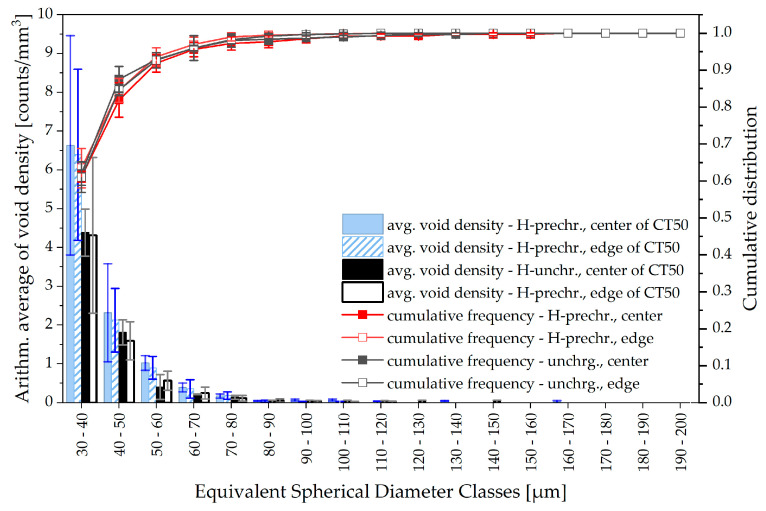
Average void densities (void count per unit sample volume) and cumulative frequencies for deformation-induced voids in ROI samples depending on the location of extraction from CT50 specimens. The analysis is based on three ROI samples (center, left edge, right edge) each extracted from two individual and independent CT50 specimens for both the uncharged and precharged conditions.

To assess whether hydrogen is more likely to induce smaller or larger voids, the cumulative frequencies of the arithmetic averages of the void count are added in Figure 10. For both conditions, about 90% of the voids have an ESD of under 50 µm. During ductile crack growth, small micro-voids typically grow to large cavities and coalesce as the crack proceeds. No deviation is visible in the cumulative frequencies between hydrogen-precharged and uncharged specimens. The consequences are discussed in the subsequent section. Figure 11, as an extension of the previous illustration of void statistics, shows that the average void density and its cumulative frequency are not affected by the extraction location of the ROI samples from the CT50 specimens.

## 4. Discussion

The results show that micro-computed tomography is highly valuable for the interpretation of hydrogen-assisted crack growth in fracture mechanical specimens. µ-CT revealed increased crack extension and a lower CTOA across the entire specimen thickness as well as an increased pore density and a tendency for secondary cracking near the main crack tip in precharged fracture mechanical samples of pressure vessel steel P355NH. Multiple crack formation during HE-associated fracture is a possible outcome of the pearlitic microstructure [22] but may also be associated with enhanced void growth and coalescence, as mentioned later.

Concerning the compact tension specimens, it is believed that the superimposition of continuous hydrogen desorption during mechanical testing noticeably affects the crack propagation. Hydrogen near the side surfaces of the samples desorbs during the J-Δa-tests, but a hydrogen reservoir may sustain at mid-thickness. Consequently, the ductile crack front near the side grooves grows in a region of lower hydrogen concentration whereas the crack at the center section extends into a regime with a higher residual hydrogen content. Following this thesis, the most pronounced effect of hydrogen on ductile crack extension, including crack morphology alterations and secondary cracking (Figure 8), is visible at mid-thickness and the influence of hydrogen on crack growth near the side grooves is weaker. Differences between ductile crack length and especially CTOA reduce near the edges of the specimen (Figure 6 and Figure 7), further confirming a potential influence of hydrogen desorption. On the other hand, a difference in void density depending on the ROI sample extraction location was not obtained. Furthermore, the through-thickness stress state, especially regarding local stress triaxiality or mechanical clamping artifacts, might also lead to crack shape deviations along the specimen thickness.

In conclusion, future research is necessary to further evaluate the influence of hydrogen desorption, featuring a comparison between precharged and in situ-charged fracture mechanical specimens. Comprehensive FEM studies, validated via experimental measurements of hydrogen permeation in P355NH, are also necessary for clarification. These simulations must accurately describe the triaxial stress field, also containing stress-assisted hydrogen diffusion and desorption. This approach, by combining 3D evaluation of local crack propagation using µ-CT with a valid simulation of hydrogen distribution, could offer significant added value compared to conventional analyses of HE. The present work provides the µ-CT measurement framework for this and the implementation of FEM analysis is planned in further studies.

It has been proposed in the past that HE, in addition to embrittling phenomena like hydrogen-enhanced decohesion (HEDE) [23], also occurs due to a modification of ductile micro-void coalescence (MVC) failure mechanisms [24]. The latter, which can be accounted to mechanisms such as hydrogen-enhanced localized plasticity (HELP) or adsorption-induced dislocation emission (AIDE) [23], can lead to finer, shallower dimples and even cleavage-like fracture morphologies such as quasi-cleavage (QC), although QC is linked to plastic processes and not to cleavage mechanisms [10]. There has also been a debate on whether hydrogen facilitates void nucleation due to increased generation of strain-induced vacancies (HESIV), therefore promoting ductile crack growth and lowering crack growth resistance, respectively [25].

Considering fractographic investigations in previous work, no clear QC-features nor alterations in the dimpled fracture morphology were found for H-precharged P355NH after the fracture mechanical tests [19]. Therefore, the HE mechanism responsible for the reduced crack growth resistance remained uncertain in the past study. On the contrary, µ-CT investigations in the present work unambiguously reveal that increased void formation due to internal hydrogen concentration at the crack is responsible for facilitated ductile crack growth (Figure 10). Some authors discuss that dimpled fracture surfaces may remain untouched even though hydrogen-assisted failure occurs or that the occurrence or absence of certain fractographic features depends on the form and localization of hydrogen-enhanced MVC processes [10,25]. Thus, it is concluded that plasticity-influencing mechanisms, in particular HESIV, HELP or AIDE, are responsible for the results obtained for hydrogen-assisted ductile crack growth in P355NH, acting simultaneously to promote localized MVC and facilitating ductile crack propagation. The CT-findings, regarding increased void nucleation, align i.a. with the results derived from [14] for hydrogen-influenced damage propagation in X70 pipeline steel.

However, the present µ-CT study does not allow for any statements on whether micro-voids in hydrogen-precharged material tend to be situated in a more localized plastic zone than in uncharged steel. Because of the remaining challenges regarding detectability and segmentation of small voids in steel using µ-CT—the minimum detectable void size in this study is 30 µm ESD—further information regarding the distribution and initiation of micro-voids is lost. It is therefore necessary to develop optimized strategies for a better imaging of very small cavities in the CT data, preferred by a combination of prolonged measurements with maximum resolution and advanced segmentation techniques. Both are to be discussed in further work. Furthermore, only the overall void density is increased in the current results because of hydrogen charging and no differences are observed for the cumulative distribution—neither at the center nor the edges of the fracture mechanical specimen (Figure 10 and Figure 11). Based on these figures, one might conclude that internal hydrogen originating from precharging seems to be responsible solely for promoted void initiation and does not influence void growth. However, very small voids, spanning only a few microns but nevertheless crucial for the understanding of early nucleation and growth processes, could not be imaged and segmented at the current stage of investigations. Higher-resolution µ-CT scans—maybe also using synchrotron radiation—and an advanced segmentation strategy may capture these small micro-void fractions, therefore representing an important direction for future research. It will also assist in clarifying whether secondary cracking or locally accelerated crack propagation is a consequence of facilitated void coalescence. Alternatively the lowered crack growth resistance as visible by means of J-integral [19] on a global and through-thickness CTOA on a local scale may only be attributed to an increase in total void density, which leads to a more rapid onset of critical ductile damage, with its original propagation mechanism being unaffected. Whether hydrogen may be responsible for enhanced void nucleation and growth or only for the first-mentioned is also discussed by [14] and the answer, just as in the present study, remains temporarily unclear.

Further investigations are also in need of in situ charging of the compact tension specimens to gain a deeper understanding of the influence of hydrogen on ductile cracking mechanisms using µ-CT. For instance, the data of precharged samples is superimposed with the effects of desorption. Nevertheless, the workflows in [19] and in the present study have proven to be valuable for a first determination of the HE susceptibility of low-alloy ferritic steels. Future µ-CT studies of hydrogen-enhanced void formation and damage propagation could also contribute to failure prediction for P355NH in hydrogen applications. Void statistics and, in particular, the void volume fraction could represent input parameters for Gurson-type ductile damage models [26].

## 5. Conclusions

The main findings of the X-ray tomographic investigation of hydrogen-assisted ductile crack growth in H-precharged pressure vessel steel P355NH are summarized in the subsequent conclusions:

X-ray micro-computed tomography revealed a pronounced surge in void density and secondary cracking at the crack tip within the hydrogen-precharged P355NH specimens. This increase in deformation-induced cavity nucleation and secondary crack paths significantly contributes to the overall degradation of the material’s ductile crack growth resistance, which is visible in both J-Δa measurements in [19] and through-thickness CTOA evaluation in the present study. To further elaborate and quantify these findings, advanced segmentation processes and in situ charging methodologies will be applied in further studies.

µ-CT data underlines the effect of superimposed hydrogen desorption on local crack propagation. Hydrogen effects near the side surfaces of the specimens seem to be reduced, possibly caused by a lower hydrogen concentration in these zones. This is depicted, for example, in CTOA as well as local crack morphology. Mechanical testing with in situ hydrogen charging will be carried out in future works for a more in-depth analysis of hydrogen-assisted cracking with limited desorption-induced effects. A comprehensive FEM analysis to obtain a valid hydrogen concentration profile is also necessary for further clarification.

Micro-computed tomography complements the fracture mechanical data, optical microscopy and SEM investigations from the previous study [19] by giving unique non-destructive three-dimensional insights in crack geometry, void distribution and damage morphology. Consequently, the hydrogen-assisted ductile crack growth could be clearly attributed to HE mechanisms like HELP, HESIV and AIDE, which was not possible using solely the 2D micrographs in [19]. The 3D characterization of damage evolution may therefore also contribute to other cases where the mechanisms involved in hydrogen-assisted damage are ambiguous. Additionally, tomographic data has the potential to be used in damage models for hydrogen-assisted crack propagation.

## Figures and Tables

**Table 1:** Scan settings for µ-CT investigations in this work using the 230 kV micro-focus tube L10802 from Hamamatsu Photonics K.K., Hamamatsu City, Japan.

Parameter Name	Set for Cracked CT50 Specimen Investigation	Set for ROI Sample Investigation
Acc. Voltage	230 kV	200 kV
Target Power	55 W	7 W
Filter	1 mm Cu	0.7 mm Cu
Detector Binning	2 × 2	2 × 2
SOD	≈170 mm	≈13 mm
SDD	≈765 mm	≈700 mm
Detector FPS	7	1.6
No. of Averages	20	15
No. of Projections	1824	1536
Voxel Size	≈55 µm	5.5 µm

**Figure A1:**
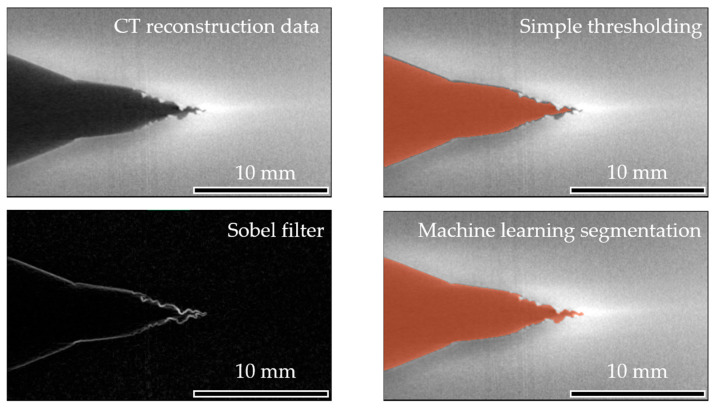
Comparison of simple thresholding and machine learning methodology for the crack segmentation in compact tension specimen reconstruction data. The simple thresholding methodology leads to inaccurate results because of beam hardening, low X-ray intensity and metal artifacts. Original CT data and slices with enhanced edge contrast using Sobel filtering are used for training the machine learning model, which shows significantly better segmentation results at the crack tip.

**Figure A2:**
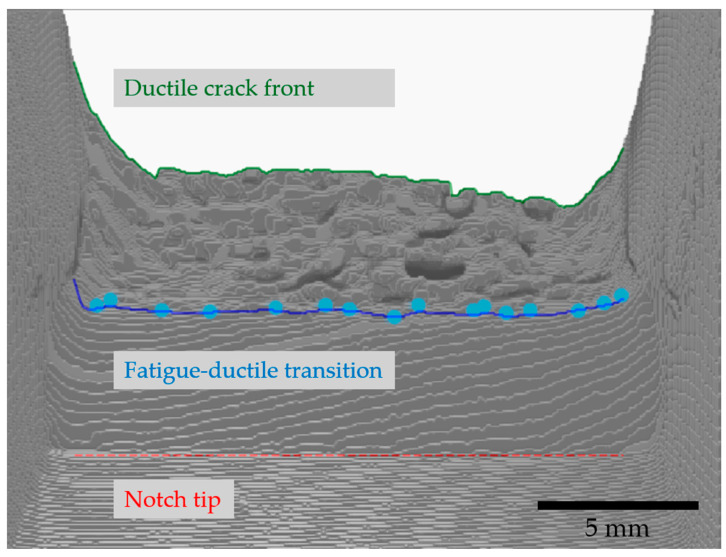
Evaluation of fatigue and ductile crack front using the Python code implemented in Dragonfly. A minimum of 15 transition points (16 in this example) is evaluated manually and the fatigue–ductile transition is interpolated using the PCHIP methodology described as above.

## Data Availability

The original contributions presented in this study are included in the article. Further inquiries can be directed to the corresponding author.

## Supplementary Materials

The following supporting information can be downloaded at https://www.mdpi.com/article/10.3390/ma19071335/s1; Figure S1: Seconardy-cracking-midthickness-1; Figure S2: Seconardy-cracking-midthickness-2.

## Abbreviations

The following abbreviations are used in this manuscript:

Δa	Ductile crack extension
AIDE	Adsorption-induced dislocation emission
CT50	Compact tension specimens with width W = 50 mm and thickness B = 25 mm
CTOA	Crack tip opening angle
EDM	Electric discharge machining
ESD	Equivalent spherical diameter
FEM	Finite element method
FIB	Focused ion beam
HE	Hydrogen embrittlement
HEDE	Hydrogen-enhanced decohesion
HELP	Hydrogen-enhanced localized plasticity
HESIV	Hydrogen-enhanced strain-induced vacancies
J	J-integral
MVC	Micro-void coalescence
PCHIP	Piecewise cubic Hermite interpolating polynomials
QC	Quasi-cleavage
ROI	Region of interest
SOD	Source-to-object distance
SEM	Scanning electron microscopy
SDD	Source-to-detector distance
SNR	Signal-to-noise ratio
µ-CT	Micro-computed tomography
