# Supplementary figures and images for: Visualizing the 3D Evolution and Morphology of Hydrogen-Assisted Ductile Crack Growth in Hydrogen-Precharged P355NH Steel Using X-Ray Micro-Computed Tomography
Source: Materials (Basel). 2026 Mar 27;19(7):1335. doi: 10.3390/ma19071335 (PMC13073654; doi:10.3390/ma19071335)

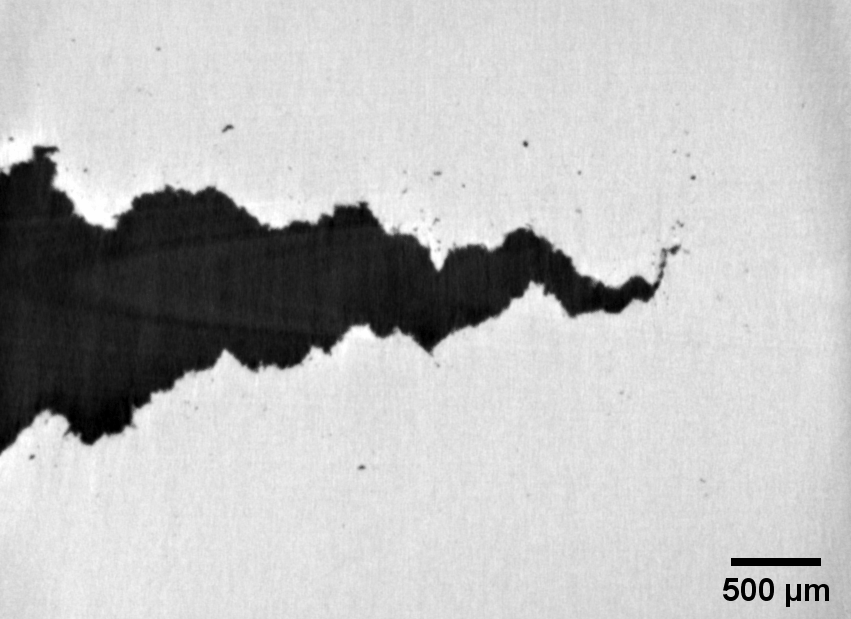

Supplement: Supplementary file 1 [file materials-19-01335-s001.zip › supplementary/Secondary-cracking-midthickness-1.gif]

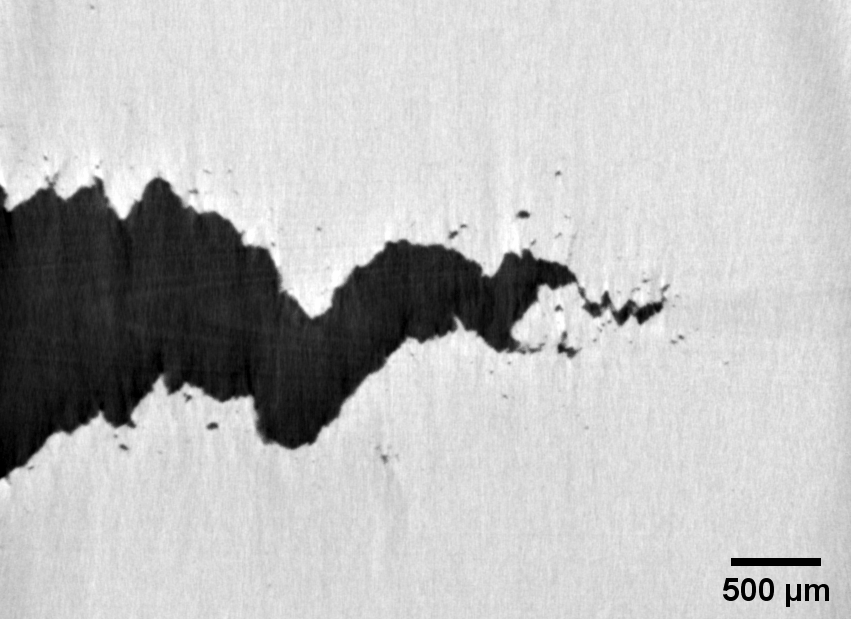

Supplement: Supplementary file 1 [file materials-19-01335-s001.zip › supplementary/Secondary-cracking-midthickness-2.gif]
